# Errata

**Published:** 2014-12-19

## Vol. 63, No. 49

In the report, “State Laws Prohibiting Sales to Minors and Indoor Use of Electronic Nicotine Delivery Systems — United States, November 2014,” on page 1149, in Figure 2, “States with and without laws prohibiting smoking and use of electronic nicotine delivery systems (ENDS) in indoor areas of private worksites, restaurants, and bars — United States, November 30, 2014,” DC was incorrectly shaded. DC should have been shaded to indicate “**Prohibits indoor smoking only**.”

In the report, “Airport Exit and Entry Screening for Ebola — August–November 10, 2014,” on page 1165, in the Figure “Number of travelers (N = 1,986*) arriving from Guinea, Liberia, and Sierra Leone who were screened for Ebola at U.S. airports, by state and county of destination — October 11–November 10, 2014,” for Minnesota, the numeral **82** should have been included in the outline for the number of travelers to that state.

In the report, “Clinical Inquiries Regarding Ebola Virus Disease Received by CDC — United States, July 9–November 15, 2014,” errors occurred in the list of authors and their affiliations. Those should read as follows:

Mateusz P. Karwowski, MD^1,2,3^, Elissa Meites, MD^2,4^, Kathleen E. Fullerton, MPH^2,5^, Ute Ströher, PhD^2,6^, Luis Lowe, MS, MPH^2,7^, Mark Rayfield, PhD^2,8^, Dianna M. Blau, DVM, PhD^2,6^, Barbara Knust, DVM^2,6^, Jacqueline Gindler, MD^2,9^, Chris Van Beneden, MD^2,10^, Stephanie R. Bialek, MD^2,11^, Paul Mead, MD^2,12^, **Alexandra M. Oster, MD^2,13^** (Author affiliations at end of text)

^1^Epidemic Intelligence Service, CDC; ^2^Epidemiology/Laboratory Task Force, 2014 Ebola Response Team, CDC; ^3^Division of Environmental Hazards and Health Effects, National Center for Environmental Health, CDC; ^4^Division of STD Prevention, National Center for HIV/AIDS, Viral Hepatitis, STD, and TB Prevention, CDC; ^5^Division of Health Informatics and Surveillance, Center for Surveillance, Epidemiology, and Laboratory Services, CDC; ^6^Division of High-Consequence Pathogens and Pathology, National Center for Emerging and Zoonotic Infectious Diseases, CDC; ^7^Division of Preparedness and Emerging Infections, National Center for Emerging and Zoonotic Infectious Diseases, CDC; ^8^Division of Global Disease Detection and Emergency Response, Center for Global Health, CDC; ^9^Global Immunization Division, Center for Global Health, CDC; ^10^Division of Bacterial Diseases, National Center for Immunization and Respiratory Diseases, CDC; ^11^Division of Viral Diseases, National Center for Immunization and Respiratory Diseases, CDC; ^12^Division of Vector-Borne Diseases, National Center for Emerging and Zoonotic Infectious Diseases, CDC; **^13^Division of HIV/AIDS Prevention, National Center for HIV/AIDS, Viral Hepatitis, STD, and TB Prevention, CDC** (Corresponding author: Mateusz (Matt) P. Karwowski, ydh4@cdc.gov, 770-488-4397)

